# A Census of Hospital Workers

**Published:** 1888-06-09

**Authors:** 


### A CENSUS OF HOSPITAL WORKERS.

It may be interesting at this time to give the following statistics of the number of persons actively employed in the various hospitals and dispensaries of London during the year 1887:—

| | |
|---|---|
| Chaplains | 40 |
| Honorary Medical Officers | 1,211 |
| Paid Medical Officers | 240 |
| Secretaries, Clerks, and Collectors | 239 |
| Matrons | 105 |
| Nurses | 1,474 |
| Stewards and Housekeepers | 56 |
| Dispensers | 148 |
| Domestie Servants (Male) | 393 |
| Domestic Servants (Female) | 664 |
| | 4,570 |

